# Staff and resident perceptions on the introduction of a team based multi-specialty resident night shift system

**DOI:** 10.1371/journal.pone.0268569

**Published:** 2022-05-19

**Authors:** Steven J. Katz

**Affiliations:** Department of Medicine, University of Alberta, Edmonton, Alberta, Canada; Yale University, UNITED STATES

## Abstract

**Objectives:**

To determine the perceptions of staff and resident physicians on the impact of implementation of a new team based multi-specialty resident night shift system.

**Methods:**

An electronic survey was distributed anonymously to all resident physicians in the Core Internal Medicine residency program at the University of Alberta. A similar survey was distributed to staff physicians in the 4 specialties impacted by this new system: hematology, respirology, nephrology and gastroenterology.

**Results:**

74 physicians completed the survey. A majority of respondents (67%) indicated the new system was a positive change. Most shared it was better than traditional 1 in 4 call (65%), with resident physicians appreciating the team based nature of the system (65%), and just more than half of residents (55%) indicating this system improved their overall wellness. Most respondents (78%) did not feel the additional handover required had a negative impact. Respondents indicated daytime teaching and feedback improved as a result of this system (52%) with most others indicating it had no impact, although overnight feedback remained a challenge.

**Conclusion:**

The implementation of this new team based system was well accepted by both staff and resident physicians across a number of domains. Future study is required to determine its impact on access and quality of care.

## Introduction

Night Float or night shift systems for residency programs are not novel [1, 2]. A system wherein instead of doing regular 24 hour or longer shifts every 3^rd^ or 4^th^ day, Night Float systems assign a resident to provide in hospital overnight coverage for a period of time, e. g. 1–2 weeks, while not working during the daytime to provide a regular period of rest. In exchange, this system decreases the amount of traditional on call required throughout the year. There is significant literature which describes the benefits, concerns and outcomes of Night Float systems, including positive effects on resident fatigue and burnout, possible equivocal effects on patient safety and medical error and an increase in patient handover. It has been adopted by many residency programs, including internal medicine [3–6].

Below is described the implementation of a unique new system locally entitled Night Pool as part of the internal medicine residency program. While the program has a night float system already in place, wherein one senior internal medicine resident covers our hospital’s general internal medicine services, this new system uniquely provides for a team of undifferentiated internal medicine residents to collectively cover multiple internal medicine subspecialty services. Six months post implementation, a survey measuring both staff physician and resident perceptions of this new arrangement was completed to determine initial opinions and beliefs of this new service.

The University of Alberta Hospital in Edmonton Canada has 4 subspecialty wards (Hematology, Respirology, Gastroenterology, and Nephrology) which were traditionally covered overnight by internal medicine residents for the past two decades. To ensure both daytime and nighttime resident in house service coverage, and to be compliant with work hour rules, residents were mandated to complete two 4 week rotations during their residency for each of these subspecialties, with 1:4 24-hour call obligations. The requirement to complete 2 rotations often limited residents’ ability to complete other subspecialty rotations over the course of their residency training. With the first priority to the ward during daytime hours, this structure also limited the amount of ambulatory clinic experiences that would occur for these specialties. Further, being away from the ward for clinics, as well as post call, interrupted daytime continuity of care and at times, disrupted staff physicians’ ability to provide performance feedback to residents as there was a perception of limited resident exposure. From a wellness perspective, this model meant residents, particularly in PGY1, were completing 1:4 overnight call coverage for over 80% of the academic year.

In 2019, the Core Internal Medicine residency program at the University of Alberta underwent a visioning exercise to review the currently offered clinical rotations for their residents. With national standards for residency training in Canada recently being updated, it put new pressures on being able to balance the educational needs of training with service requirements traditionally provided by the residency program [7, 8]. Specifically, with the introduction of Competency Based Medical Education, other residency programs re-evaluated their requirements to have their residents complete rotations in Internal Medicine, thereby reducing the available resident workforce for medicine wards, and new standards strongly suggested earlier exposure to ambulatory settings for internal medicine residents. New local standards became more restrictive with regards to on call eligibility for residents and there was a changing relationship with the local health authority [9].

In order to address these concerns, nighttime coverage and ambulatory clinics were separated from the main specialty rotations. Instead, residents rotating through these rotations, now required for only 1 4-week block, are entirely available to provide daytime in house coverage Monday to Friday. They are asked to stay late 1–2 days per week to cover a gap between when the daytime team ends and the nighttime team starts, and then also complete a traditional call shift on one Friday night, 24 hours on Saturday and daytime Sunday, during a 4-week rotation.

To provide nighttime coverage, a new rotation was introduced called Night Pool. Night Pool is a 2 week 12-hour shift based overnight rotation which runs Sunday night to Thursday night. A minimum of four residents are on this rotation at any given time. Each resident is assigned as a lead for one of the four subspecialties, ideally having completed a daytime rotation previously; and ideally serving as a lead for at least two different specialties during the rotation. The lead resident is responsible for receiving and providing handover at the beginning and end of the shift respectively to the daytime team, and will be first to respond to ward concerns as well as new consults for the service. Uniquely to Night Pool, a resident who is not busy with their primary service is expected to help another Night Pool resident should their service be busy, or alternatively may assist the Night Float general medicine senior resident. Further, if a patient requires the medical opinion of more than one of these specialties, only one resident from the team assesses the patient, but discusses their findings and management plan with the staff from both specialties. The idea is to create efficiencies, encourage better distribution of workload amongst the residents and foster a team approach to care and resident wellness. If a resident does assist the lead resident, they are responsible to update the lead resident on any issues to safeguard appropriate handover at the end of the shift.

## Methods

Six months after implementation of the Night Pool system described above, a survey was sent to all residents in the internal medicine program as well as specialty supervisors—subspecialty staff and subspecialty senior residents—to determine their perceptions of this new system compared to the traditional system (See Table 1A and 1B). At the time, a survey was pursued to ensure the new system was achieving its intended objectives, was acceptable to the stakeholders and had no unintended consequences. The survey was sent directly by the program to the residents, whereas the survey link was shared with subspecialty administrators to share with their teaching staff. Below are the responses provided by the supervisor physicians and residents.

**Table 1:** Night pool perception survey questions.

A–Residents Questions:
1. Overall, Night Pool was: [1 = very poor, 5 = excellent]
2. Did Night Pool provide a more team approach or create more efficiencies to work than before [1 = No, 3 = No difference, 5 = Yes!]
3. How does Night Pool compare to 1:4 Call on subspecialty service? [1 = prefer 1:4 call, 5 = prefer Night Pool]
4. For PGY1s, did you feel prepared for Night Pool?
5. Night Pool results in an extra handover. Was handover: [1 = very poor, 5 = excellent]
6. Is it important to complete the daytime specialty rotation prior to providing overnight coverage?
7. If you completed a subspecialty rotation, what did you think about late stay call? [1 = very poor, 5 = excellent]
8. Do you prefer late stay and weekend call, or traditional 1:4 call?
9. Over the course of the last 6 months, Night Pool and the change related to subspecialty call impacted my overall wellness: [1 = Negatively, 3 = no difference, 5 = Positively]
10. Do you think the changes made [Night Pool, minimal call on specialty rotations] impacted your learning of these specialties? [1 = Yes, learned much better than I would have under the old system, 3 = No impact, 5 = learned much worse than I would have under the old system]
11. What impact did not being post call on your daytime specialty service have on your ability to receive feedback on your work? [Select all that apply]
12. What impact did Night Pool have on your ability to receive feedback on your work? [Select all that apply]
B–Staff Questions:
1. Your Specialty:
2. Are you staff or a subspecialty resident?
3. Overall, Night Pool was: [1 = very poor, 5 = excellent]
4. How does Night Pool compare to 1:4 Call on subspecialty service? [1 = prefer 1:4 call, 5 = prefer Night Pool]
5. Did you find the Night Pool residents were more or less prepared [competent at an expected level] for their clinical duties compared to 1:4 call? [1 = less prepared, 3 = same, 5 = more prepared]
6. Do you think it is important to complete the daytime specialty rotation prior to providing overnight coverage?
7. How do you think the late stay on call time [evening 5-9pm] went? [1 = very poor, 5 = excellent]
8. Based on your experience, would you prefer the daytime residents continue doing late stay and weekend call, or return to a traditional 1:4 on call model?
9. Daytime residents are no longer post call, meaning they are present more often for clinical work. With regard to clinical care, do you see this negatively [1], positively [5], or has made no difference [3]?
10. Night Pool results in an extra handover. Was handover: [1 = very poor, 5 = excellent]
11. Do you think the changes made [Night Pool, minimal call on specialty rotations] impacted learning by the daytime residents of these specialties? [1 = learned much worse than under the old system, 3 = No impact, 5 = learned much better than under the old system]
12. What impact did Night Pool have on your ability to provide feedback on the work of the overnight team? (provided the same feedback on work vs provided less regular feedback on work)
13. What impact did Night Pool have on your ability to provide feedback on the work of the day team? (provided more feedback on work vs provided the same feedback on work)

Ethics approval was waived by the University of Alberta Ethics Board as the survey was completed as part of a Quality Assurance project for this innovation.

## Results

Forty-eight of 107 eligible internal medicine residents [45%] and 26 specialty supervisors (denominator unknown as described in the methods above) completed the survey. Of the residents, 15 were first year residents (31%), 19 were second year residents (40%) and 14 were third year residents (29%). From the supervisor survey, 9 were subspecialty residents with the remaining staff physicians; 11 were from nephrology (42%), 6 respirology (23%), 4 gastroenterology (16%) and 5 hematology (19%). Result highlights can be found in Table 2.

**Table 2:** Highlighted night pool survey results [%].

	**Overall Impression of Night Pool**					
	1 [Very Poor]	2	3	4	5 [Excellent]	N/A
Staff	0	0	15.4	53.8	30.8	0
Residents	2.1	6.3	27.1	29.2	14.6	20.8
	**Did Night Pool provide a more team based approach or create more efficiencies to work than the previous system?**					
	1 [No]	2	3 [No difference]	4	5 [Yes]	N/A
Residents	2.1	4.2	14.6	27.1	37.5	14.6
	**How does Night Pool compare to 1:4 Call on subspecialty service?**					
	1 [Prefer 1:4 Call]	2	3	4	5 [Prefer Night Pool]	N/A
Staff	11.5	3.8	7.7	38.5	38.5	
Residents	8.3	8.3	8.3	25	33.3	16.7
	**Did you feel prepared for Night Pool [applicable to PGY1 residents only]?**					
	Yes	No	Sometimes	N/A [PGY2/PGY3]		
Residents	8.3	2.1	18.8	70.8		
	**Did you find the Night Pool residents were more or less prepared [competent at an expected level] for their clinical duties compared to 1:4 call?**					
	1 [less prepared]	2	3 [No Change]	4	5 [more prepared]	
Staff	3.8	11.5	38.5	34.6	11.5	
	**Night Pool results in an extra handover. Was handover:**					
	1 [Poor]	2	3 [no change]	4	5 [Excellent]	
Residents	6.3	16.7	45.8	18.8	12.5	
Staff	3.8	15.4	57.7	11.5	11.5	
	**Over the course of the last 6 months, Night Pool and the change related to subspecialty call impacted my overall wellness:**					
	1 [Negatively]	2	3 [No Change]	4	5 [Positively]	N/A
Residents	2.1	16.7	16.7	25	29.2	10.4
	**Do you think the changes made [Night Pool, minimal call on specialty rotations] impacted learning of these specialties?**					
	1 [Worse]	2	3 [No Change]	4	5 [Better]	N/A
Residents	2.1	8.3	35.4	14.6	29.2	10.4
Staff	0	0	30.8	34.6	34.6	

When both residents and staff groups were asked their overall impression of Night Pool, 14.6% residents and 30.8% staff rated it as 5 on a 5 point Likert scale, 29.2% and 53.8% as 4, 27.1% and 15.4% as 3, and 8.4% and 0% as two or less. 20.8% of residents [N = 10] did not share an opinion as they had not yet completed a Night Pool rotation.

Residents felt Night Pool had a beneficial team based approach compared to the previous system, with 37.5% of residents rating the new system as a 5, 27.1% as a 4, 14.6% as a 3, and 6.3% as two or less, indicating they felt the previous system was better. Similarly, 58.3% of residents liked this system better than traditional 1:4 call, while only 16.6% likely the previous system better. Over 80% of staff also felt this system was superior to traditional 1:4 call, with 15.3% indicating they liked the traditional system better.

PGY1 residents who had not experienced the previous traditional 1:4 system were asked if they felt prepared to do Night Pool. Out of 15 respondents, 4 said yes, 1 no and the remaining said sometimes, with many commenting it would likely be hard to do something new the first time regardless. Nearly 85% of staff respondents felt residents were at least as well prepared, if not better, than the previous system.

Residents were asked about the impact of an additional handover created by the new system. 45.8% of residents felt it had no impact (30 with the remaining split evenly between having a positive or negative impact. Staff felt similarly, with 57.7% feeling it had no impact, and 23% feeling handover was superior compared to the old system.

Residents were asked what they thought about the new late stay shift when completing a daytime rotation. 27.1% of residents thought it was excellent (5), 29.2% rated it as a 4, with only 16.7% indicating it was poor or very poor. 75% of residents prefer the new system requiring late stay and weekend call compared to 1:4 call. Similarly, all staff felt these shifts went well, with no one rating it below a 3, and a 92.3% preference compared to the traditional system.

With regards to resident wellness, 54% of residents thought their overall wellness had improved as a result of the introduction of the new system, 16.7% indicating there was no change, with 18.8% indicating they have been negatively impacted.

Both staff and residents felt the changes improved daytime learning for those completing the specialty rotations. Nearly 70% of staff thought learning improved with the remaining noting no change, while 43.8% of residents felt learning improved, 35.4% thought there was not difference and 10.4% thought learning was worse. Residents and staff thought there was also a small increase in providing residents with feedback on their clinical work during their daytime shifts compared to before, but less feedback being provided on overnight work by the Night Pool team.

## Discussion

The internal medicine residency program successfully implemented a team based multi-specialty overnight shift system, the first system like this described in the literature. The feedback provided after the initial implementation by both staff and residents is encouraging: Both groups shared a positive initial impression of this change, and both felt it was superior to the traditional 1:4 on call system. Similarly, both groups felt the late stay system was superior to the traditional 1:4 call, and neither group felt the addition of an extra handover to facilitate this new system was problematic. Residents generally felt the new system positively impacted their overall wellness, and both groups indicated that daytime learning improved as a result of improved resident presence.

These results are perhaps not surprising based on the available literature on similar systems [10]. Others have previously demonstrated positive overall perceptions of similar night float systems [1, 2, 10], although this is the first reported system which incorporates the concept of a resident overnight team covering multiple specialties simultaneously. One of the previous concerns around these systems is a deficit in formal teaching during the night [3–5], and the experience here was no different in that residents perceived they received less feedback on overnight work. However, this survey also demonstrated a perceived improvement in learning during daytime shifts as a result of the new system’s impact on the subspecialty rotations. As well, this survey demonstrated clear staff preference for this new model, which is different than some previous studies [11].

The acceptance of this system by residents and staff should create work efficiencies that benefit not only the residents, as they perceive from the survey results, but potentially patient care. Future reviews of this new format should investigate the impact on patient wait times for care, with the hypothesis being that wait times decrease due to the shared team responsibility to provide care; as well as quality care measures for both the daytime and nighttime shifts.

This is important as the results in this survey do not demonstrate whether or not the quality of care has changed, both during the daytime, where housestaff continuity of care is improved, or at night. Other studies suggest quality of care is at least just as good [11–13], although these examined a more traditional night float model rather than the multi-specialty model described here. These results also do not address whether this impacts the volume and breadth of cases residents may see as a result of these changes, although previous studies have not demonstrated concerns in this regard [6, 14–16].

It is also unclear if the results of the survey are truly representative of the overall resident experience, although a high survey response rate of 45% is reassuring. It is also possible that the Night Pool system has different impact or benefits on subgroups of residents. Future studies collecting resident gender, age and other demographic information may provide further clarity. As the program already has a Night Float system, it is also unclear what influence the experience of a prior similar system had on the implementation of the Night Pool system.

It is also unclear if the results are representative of the staff experience. Due to the survey distribution method, the proportion of staff who chose to complete the survey is unknown, and whether or not the results are representative of the larger group. Response numbers for each subspecialty were small; it is possible that the Night Pool system works better for certain subspecialties over others and the data does not capture this. Future surveys as more experience is gained in this new system may also help determine whether or not the initial benefits are sustained over time. Opportunities to implement and study this system in other settings are also needed to ensure the results are generalizable.

In conclusion, the internal medicine residency program successfully introduced a novel team based multi-specialty overnight shift system which was well accepted by both supervisor physicians and residents. Further study is necessary to explore all its impact, but this system may be worth consideration by other institutions with similar challenges.

## Supporting information

S1 Data (XLSX)
